# Comparison of dental caries (DMFT and DMFS indices) between asthmatic patients and control group in Iran: a meta-analysis

**DOI:** 10.1186/s40733-021-00068-y

**Published:** 2021-02-04

**Authors:** Nadia Elyassi Gorji, Pegah Nasiri, Ali Malekzadeh Shafaroudi, Mahmood Moosazadeh

**Affiliations:** 1 Dentistry Student, Student Research Committee, Faculty of Dentistry, Mazandaran University of Medical Sciences, Sari, Iran; 2 Gastrointestinal Cancer Research Center, Non-communicable Diseases Institute, Mazandaran University of Medical Sciences, Sari, Iran; 3 Health Science Research Center, Addiction Institute, Mazandaran University of Medical Sciences, Sari, Iran

**Keywords:** Asthma, DMFT index, Dmft index, DMFS index, Dental caries

## Abstract

**Background:**

The association between caries index, which is diagnosed by Decayed, Missing, and Filled Teeth (DMFT), and asthma has been assessed in several studies, which yielded contradictory results. Meta-analysis is the statistical procedure for combining data from multiple studies and reducing the differences among parameters due to the increased number of studies involved in the analysis process. Therefore, the present study aimed to determine the relationship between dental caries using decayed, missing, filled teeth indices (DMFT, dmft, and DMFS indices) and asthma using meta-analysis.

**Methods:**

Databases were searched using such keywords as “Asthma,” “Caries,” “DMFT,” “DMFS,” “Iran,” and OR operators, AND, and NOT. After the elimination of duplicate documentation, the articles which met the inclusion criteria were selected. Quality assessment was performed based on the Newcastle-Ottawa Quality Checklist (NOS). After that, standardized mean difference (SMD) of DMFT, dmft, and Decayed, Missing, and Filled Surfaces (DMFS) indices were estimated.

**Results:**

The number of 10 evidence was extracted out of nine studies in which mean oral health indices were compared between asthmatic patients and the control group. Out of 10 evidences that examined the association of DMFT, dmft, and DMFS with asthma, these indices were higher in asthmatic patients than the control group in seven cases. In three cases, these differences were statistically significant. The SMD of DMFT, dmft, and DMFS indices between asthmatic patients and the control group at the confidence level of 95% were reported as 0.29 (− 0.05, 0.62), 0.48 (− 0.20, 1.17), and − 0.05(− 0.30, 0.21), respectively.

**Conclusion:**

According to the results, the prevalence of dental caries is higher among patients with asthma than in the control group. Therefore, having asthma could be considered a risk factor for the development of dental caries.

## Introduction

Oral health is one of the crucial factors that contribute to people’s general health [1]. The DMFT index (Decay-missing-filled teeth index) is one of the best epidemiological indices in dentistry that reflects oral and dental health in the community. It is also known as the caries index [2]. It was proposed by Klein et al. in 1938 as an index of decayed, filled, and missed permanent teeth to evaluate the prevalence of coronal caries [3].

Although oral diseases threaten all age and sex groups, some community groups are more vulnerable due to specific physiological conditions. For instance, asthma is a chronic respiratory syndrome that causes inflammation, irritability, and stenosis (spasm) of the lung’s airways [4]. This disease is characterized by the infiltration of mast cells, eosinophils, and lymphocytes, which results in airway hypersensitivity, mucosal edema, and mucus production [5]. General symptoms of asthma include wheezing, coughing, and shortness of breath [6]. According to epidemiological studies, this disease’s incidence has increased over the past two decades [7].

According to a Global Asthma Network (GAN) report in 2014, over 300 million people are infected with asthma, and this number is increasing by the day [8]. According to statistics published in 2007, the prevalence of asthma in Iran was 13.14%, which is higher than the world average [9]. Considering this significant increase in asthma prevalence, it seems that environmental factors linked to modern lifestyle contribute significantly to this disease’s etiology [10]. Although this disease has no definitive cure, it can be controlled in most patients using preventive measures and appropriate drug interventions (e.g., β2 agonists, inhaled bronchodilators, inhaled corticosteroids, and sodium cromoglycate). Inhaled corticosteroids (ICSs) are the standard treatment for most patients, and in adults who do not respond to ICSs, long-acting β2 agonists are used along with ICS [11, 12].

Several studies have been conducted on the relationship between asthma and oral health; nonetheless, they have yielded contradictory results. Some of these findings suggested a positive association between asthma and caries [13–15] while, some others did not report such a relationship and believed that no significant difference would be found in caries prevalence between asthmatic patients and healthy individuals if they observe dental care [16–18].

It has also been suggested that patients with asthma are more susceptible to caries progression since anti-asthmatic drugs have a relatively low pH [19] and can contain sweeteners and fermentable carbohydrates, such as lactose monohydrate [20, 21]. Moreover, these medications affect the salivary flow rate [22], increasing the susceptibility of people with asthma to caries. Also, patients with asthma may be more likely to use erosive beverages [23], and their mouth breathing habit may contribute to more decay in these individuals [24]. Most asthmatic patients use inhaled drugs in the wrong way; in other words, instead of the upper airway, large amounts of medication are administered in the oral cavity, which potentially has decaying effects [25].

Therefore, regarding the findings mentioned above and the inconsistencies in the existing knowledge of the relationship between oral health indices and asthma, the present study aimed to estimate the relationship between dental caries using decayed, missing, filled teeth indices (DMFT, dmft, and DMFS indices) and asthma using meta-analysis and to determine the standardized mean difference (SMD) of caries indices between patients with asthma and the control group.

## Methods

### Inclusion and exclusion criteria

Inclusion and exclusion criteria were determined according to the PI(E)CO framework (population, exposure, comparator, and outcome). In the present study, “P” includes patients with asthma among the primary studies, “I (E)” means exposure to asthma. “C” stands for the control group, which included patients without asthma during the primary studies, “O” contains SMD of DMFT, dmft and Decayed, Missing, and Filled Surfaces (DMFS) indices in the asthmatic and control groups [26].

The included articles included case-control and historical cohort studies. Moreover, studies published from any time until the end of February 2020 were also included in the inclusion criteria. The searching time was March 27, 2020, and the articles were published in English and Persian. It is worthy to note that the preliminary studies were conducted in Iran.

### Search strategy

We followed the Preferred Reporting Items for Systematic Reviews and Meta-Analyses (PRISMA) guidelines for study design, search protocol, screening, and reporting. PubMed, Scopus, Science Direct, Web of Science databases, and Iranian databases, such as Magiran and SID were searched using such keywords as “Asthma,” “Caries,” “DMFT Index,” “DMFS Index,” “Iran,” and OR operators, AND and NOT.

The search strategy in the PubMed database was as follows:

The list of published study sources was reviewed to increase the sensitivity of the search and select more studies. Endnote software was used to manage resources.

### Selection of studies

Duplicate documentation was initially removed due to overlap of content in different search databases, and non-relevant articles were subsequently excluded based on the title and abstract of the initial screening studies. After that, full-text articles were received by two people to check the inclusion and exclusion criteria, and the inconsistencies were discussed.

### Data extraction

The data were extracted independently by two individuals based on the format mentioned in the methods section. The third author was responsible for matching the two extracted files, but no disagreement was not found. Extracted variables included first author’s family name, article title, journal name, year of publication, place of study, the sample size in asthmatic patients, sample size in the control group, mean and standard deviation of DMFT, dmft, and DMFS parameters, method of selection of the cases and controls, matching the case and control groups by age and sex, and evaluation of DMFT, dmft, and DMFS indices in the case and control groups.

### Quality assessment

The quality assessment of the initial articles which met the inclusion criteria was carried out using the Newcastle-Ottawa Scale (NOS) checklist. The checklist score is between 9 and 0 [27]. Two individuals independently performed the quality assessment, and articles with a score of less than five were excluded.

### Statistical methods

The obtained data were analyzed in Stata software (version 11). The heterogeneity index between studies was determined using the I2 test. The random-effect model was used to estimate the standardized difference of mean DMFT, dmft, and DMFS parameters in the asthmatic group, compared to the control group. It should be noted that the inverse variance method was used to estimate the association between the mentioned indices with asthma. Forest plot, which is typically used to present meta-analysis results, was applied to calculate point estimates of standardized mean difference of DMFT, dmft, and DMFS indices with a 95% confidence interval. In the forest plot, the size of the square represents the weight of each study, and the lines on both sides depict a 95% confidence interval. Also, each initial study’s impact on the overall estimate was assessed by sensitivity analysis. Statistical significance was based on the mean difference’s confidence interval, which if the mean difference’s confidence interval included zero, the differences were not significant. If it did not include zero, the differences were considered significant.

## Results

**Fig. 1:**
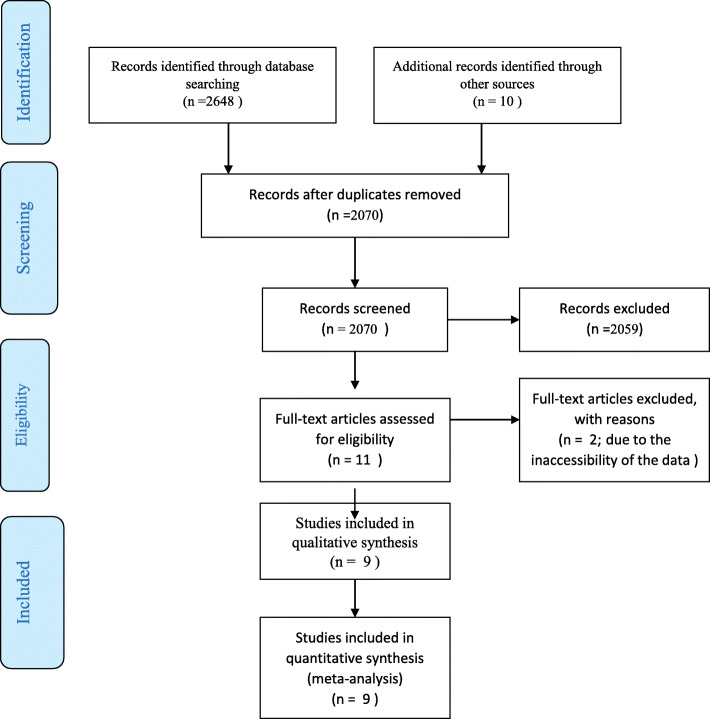
Searching and selection of the primary studies

A total of 2658 articles were identified by searching the various databases based on the method section’s strategy. Duplicate documentation was removed due to overlapping databases. Subsequently, full-texts of 11 articles were reviewed, out of which two papers were excluded due to the inaccessibility of the data. Finally, nine articles were evaluated for quality, all of which met the minimum inclusion criteria (Fig. 1) [28–36].

**Fig. 2:**
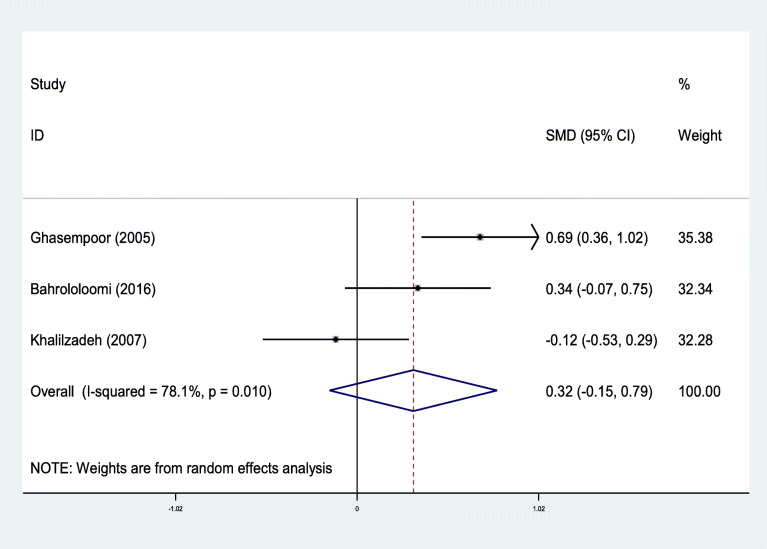
Standard mean difference forest plots of **DMFT** Index in each primary study and its overall estimate

**Fig. 3:**
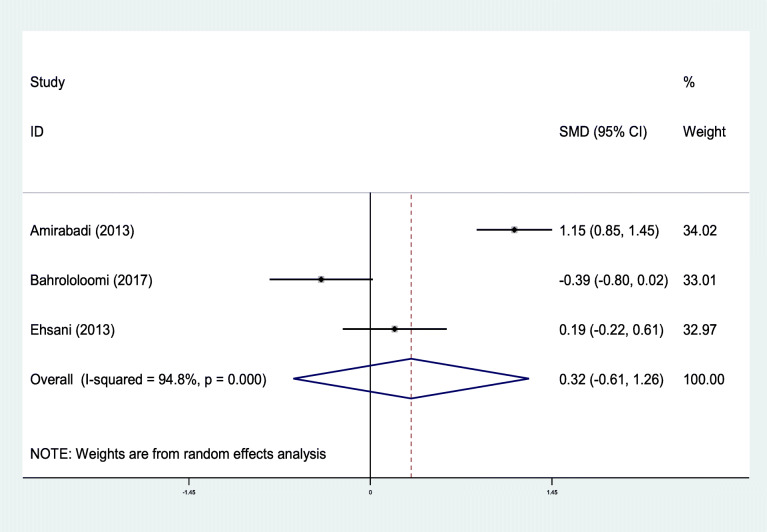
Standard mean difference forest plots of **dmft** index in each primary study and its overall estimate

Out of nine studies that compared the mean of oral health indices of asthmatic patients to the control group, ten pieces of evidence were extracted (Table 1). Out of 10 evidence that examined the relationship of DMFT, dmft, and DMFS with asthma, in 7 cases, these indices were higher in asthmatic patients than the control group [28, 29, 31, 32, 35, 36]. In three cases, these differences were statistically significant [28, 31, 36]. Since the investigated indices’ nature varied among the preliminary studies included in the meta-analysis, these studies’ results were separately combined according to DMFT, dmft, and DMFS. The relationship between DMFT and asthma was examined in 4 out of 10 evidence. These four studies’ mixed results indicated that the mean standardized difference with a 95% confidence interval was estimated to be 0.29 (− 0.05, 0.62). It is worthy to note that the heterogeneity of the results of the preliminary studies was significant (I-squared: 69.9%, Q: 9.95, *P* = 0.019) (Fig. 2). Moreover, in 4 of 10 evidence that examined the association between dmft and asthma, the standardized mean difference with a 95% confidence interval was estimated to be 0.48 (− 0.20, 1.17). There was also a high heterogeneity between the results of these four studies (I-squared: 93.0%, Q: 42.69, *P* < 0.0001) (Fig. 3).

**Table 1:** Characteristics of primary studies compiled to meta-analysis: the association between oral hygiene indices and asthma

First author, publication year	Area study	Type study	Case (Asthma)			Control			Score of quality assessment
			Sample size	Mean	SD	Sample size	Mean	SD	
**Ghasempoor 2005** [36]^a^	Babol	Case-control	75	2.27	2.65	75	0.8	1.41	7
**Bahrololoomi 2016** [29]^a^	Yazd	Case-control	46	0.9	0.691	47	0.6	1.027	8
**Khalilzadeh 2007** [33]^a^	Tehran	Case-control	45	3.98	2.53	46	4.30	2.81	6
**Hassanpour 2019** [31]^a^	Sabsevar	Case-control	70	0.71	1.47	70	0.48	1.002	7
**Amirabadi 2013** [28]^b^	Zahedan	Case-control	100	3.1	2.1	100	1.14	1.2	7
**Bahrololoomi 2017** [30]^b^	Yazd	Case-control	46	4.15	3.27	47	5.25	2.25	8
**Ehsani 2013** [32]^b^	Tehran	Case-control	44	3.34	1.71	46	3.0	1.8	7
**Hassanpour 2019** [31]^b^	Sabsevar	Case-control	70	3.79	1.83	70	2.32	1.21	7
**Salem 2018** [35]^c^	Rasht	Historical cohort	73	0.87	0.34	73	0.87	0.32	8
**Salem 2009** [34]^c^	Tehran	Case-control	45	3.98	2.53	46	4.30	2.81	8

^a^ **DMFT** included to meta-analysis

^b^ **dmft** included to meta-analysis

^c^ **DMFS** included to meta-analysis

**Fig. 4:**
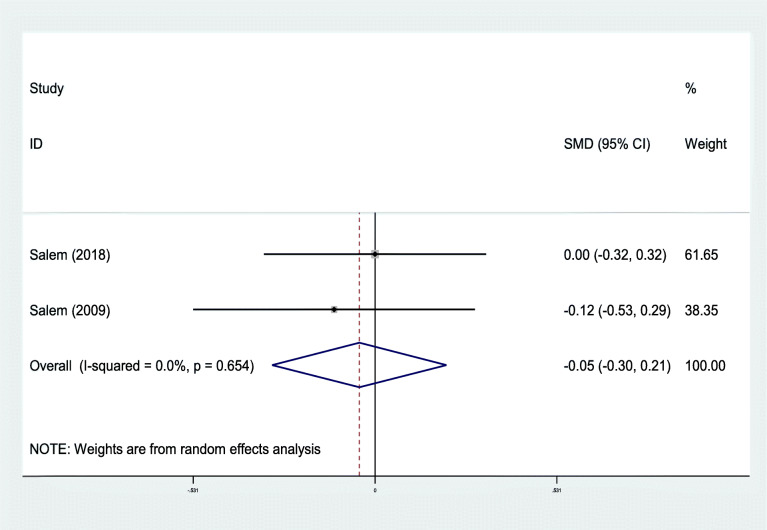
Standard mean difference forest plots of **DMFS** Index in each primary study and its overall estimate

In 2 out of 10 evidence that examined DMFS and asthma association, the standardized mean difference with a 95% confidence interval was calculated as − 0.05 (− 0.30, 0.21). The heterogeneity between these two studies’ results was low (I-squared: 0.0%, Q: 0.20, *P* = 0.654) (Fig. 4). Based on the sensitivity analysis results, each study’s effect on the overall estimate was not significant.

## Discussion

In the present study, to investigate the association of asthma with dental caries (measured by DMFT, dmft, and DMFS indices), the results of preliminary studies were combined using meta-analysis. The obtained results indicated that DMFT and dmft indices in patients with asthma were 0.29 and 0.48 more than the control group, respectively. Although this difference is not statistically significant, it is clinically significant. Also, the DMFS index in patients with asthma was 0.05 less than the control group, which was not statistically significant.

In a meta-analysis in 2011, Alavaikko et al. investigated 18 studies on asthma and dental caries in deciduous teeth (from 1950 to May 2010). Consistent with the present study result, they reported that patients with asthma are at significantly greater odds for experiencing caries [37]. Kargul et al. concluded that during the first 30 min of inhalation, the salivary pH decreased significantly. The plaque pH (pH = 5.5) dropped to a pH lower than required enamel demineralization [38]. Also, Ersin et al. reported that prolonged use of inhaled drugs lowers PH and reduces salivary flow leading to the increased colonization of streptococcus mutans and lactobacillus, which play a significant role in the development of dental caries [22]. In contrast to the two mentioned studies, a study performed by Wogelius et al. on 5 to 7-year-old children demonstrated that the administration of anti-asthmatic drugs did not lead to an increased prevalence of caries [39]. Moreover, Brigic et al. did not find the use of inhaled drugs (IADs) to be associated with decreased saliva and increased colonization of cariogenic bacteria [40].

In patients with asthma, due to reduced oxygen supply to active ameloblasts, enamel formation is impaired, which results in enamel defects. Since ameloblast cells are highly susceptible to systemic and genetic abnormalities, they cannot repair after injury [38]. Consequently, respiratory diseases in the early years of life can affect tooth formation and contribute to the development of hypo mineralized enamel lesions that affect DMFS [38, 41, 42]. Also, the concentration of nitric oxide increases in the exhaled breath of people with airway inflammatory diseases. In water, nitrous oxide converts to nitric acid, and since the oral cavity is very moist, the risk of demineralization of hard dental tissues increases [43]. Also, it is noteworthy that genes related to the immune cell signaling pathway are expressed differently in people with asthma [44] and may be involved in the association between asthma and high risk of caries. About 30 single nucleotide polymorphisms (SNPs) are known to be associated with asthma [45]. Out of the genes identified by SNP, *SPRY1/ANKRD50* and *SLC7A11/PCDH18* genes in chromosome 4 which are located approximately 70 million base pairs from AMBN (Ameloblastin) and IL2RB gene in chromosome 22, which are located about 11 million base pairs from (Tuftelin interacting protein 11) TFIP11 are involved in the development of dental caries in children with asthma. The *AMBN rs4694075* gene that controls enamel crystals’ elongation process and contributes to enamel mineralization during tooth development is involved in the initiation of dental caries in patients with asthma [46]. Another gene is *CD-14,* which is indicated as an example of gene-environment correlation in asthma [47], and genes encoding beta-defensin 1 [48] and matrix metalloproteinases [49] may also be involved in the relationship between caries and asthma. Therefore, a theory is proposed that the increased prevalence of caries in people with asthma may also be due to genetic variation in enamel formation genes [50–52].

The immune response also plays a role in caries-related outcomes in patients with asthma so that higher levels of IgG are against *Streptococcus mutans* and *lactobacillus*. Since serum IgG levels in asthmatic patients are more elevated than non-asthmatic patients [53], elevated serum IgG, and IgA levels in asthmatic patients may be attributed to the use of inhaled corticosteroids [54]. On the other hand, Galaviz et al. suggested a reverse association between asthma and dental caries, and the prevalence of dental caries is lower in patients with asthma [55]. According to studies conducted in this field, apart from the disease’s pathophysiological changes, oral changes in patients with asthma can also be due to medication. Inhaled anti-asthma drugs can make asthmatic patients more susceptible to dental caries due to decaying compounds, unpleasant taste, the effect on flow, PH, and the salivary buffering capacity, as well as the systemic effects [25, 56, 57].

Although most patients use a multi-drug regimen, a study conducted by Heidari et al. found that among the three types of anti-asthmatic medications (spray, syrup, and tablets), tablets are more likely to cause caries [58]. Other studies have indicated that syrup forms of the drug (due to sugar and high concentration) [59] and spray (from 60% for powdered inhalation drugs to 80% for aerosol metered-dose medicines which remain in the oral cavity after administration) [60, 61] are more effective in an increased risk of caries. A study carried out by Ersin et al. also demonstrated that the type of anti-asthmatic medication did not affect salivary flow rate, pH, and salivary buffering capacity [22]. Salem et al. also indicated that the dual-drug regimen of β2 agonists and corticosteroids (bronchodilator with anti-inflammatory) reduce the severity and frequency of asthma attacks. Therefore, the patient is less in need of β2 agonists, a potent salivary-reducing agent, reducing the risk of caries in patients [37].

Based on the current meta-analysis results and existing knowledge of the association between DMFT parameters and asthma, it is suggested that people with asthma undergo frequent dental examinations more frequently than healthy individuals. Therefore, it is recommended that some measures be taken to raise their awareness of the factors affecting dental decay.

They should also be instructed on the correct use of inhaled drugs; moreover, the application of inhalation spacer (which facilitates oral inhaler administration) is suggested to prevent the spread of drugs in the oral cavity. Furthermore, patients should be instructed to rinse their mouths with neutralizing or pH balancing mouthwash (such as sodium fluoride) immediately after using inhaled drugs. In children with asthma, preventive measures, such as regular fluoride therapy, fissure sealants of cavity-prone teeth, and everyday mouthwash use, should be taken from the outset. A pulmonologist should be consulted on the possible change in drug composition (recommending dual drug regimens) or training on single-drug side-effect reduction strategies.

### Limitations

Every study has some limitations which should be addressed in the paper. The possible limitation of this meta-analysis is related to the diversity of the included preliminary studies. The study population of preliminary studies may have been different in terms of such variables as genetics, ethnicity, nutritional status, general literacy level, health literacy level, socioeconomic status, and access to oral health. Due to the inability to extract variables such as genetics, ethnicity, economic and social issues from the initial studies, it was impossible to evaluate them by meta-regression.

The studies reviewed in this meta-analysis did not mention whether the asthmatic patients they examined were current or former asthmatics. Therefore, the asthmatic category mentioned in this meta-analysis might have included both current and former asthmatic patients. This can be listed as one of the limitations of this study as we cannot answer this question according to the evidence extracted from the reviewed articles.

## Conclusion

This meta-analysis demonstrated that the DMFT and dmft indices’ status was higher in asthmatic patients than in the control group. Although the DMFS index was lower in asthmatic patients, this finding wasn’t statistically significant. Therefore, it could be concluded that asthmatic patients had a higher prevalence of dental caries than people without asthma.

## Data Availability

The datasets used and/or analyzed during the current study are available from the corresponding author on reasonable request.

## Abbreviations

DMFT: Decay-Missing-Filled Teeth (for Permanent Teeth)
dmft: Decay-missing-filled teeth (for primary teeth)
DMFS: Decay-Missing-Filled Surfaces (for Permanent Teeth)
NOS: Newcastle-Ottawa Scale
GAN: Global Asthma Network
ICSs: Inhaled corticosteroids
PICO: Population, Intervention, Comparator, and Outcome
IADs: Inhaled Anti-asthmatic Drugs
SNPs: Single Nucleotide Polymorphisms

## References

[1] Ekhtiari YS, Majlessi F, Foroushani AR, Shakibazadeh E (2014). Effect of a self-care educational program based on the health belief model on reducing low birth weight among pregnant Iranian women. Int J Prev Med.

[2] Sajadi FS, Mosharafian S, Torabi M, Hajmohamadi S (2014). Evaluation of DMFT index and significant caries index in 12-year-old students in Sirjan, Kerman. J Isfahan Dent Sch.

[3] Laajala A, Pesonen P, Anttonen V, Laitala ML (2019). Association of enamel caries lesions with oral hygiene and DMFT among adults. Caries Res.

[4] Kudo M, Ishigatsubo Y, Aoki I (2013). Pathology of asthma. Front Microbiol.

[5] Brightling CE, Bradding P, Symon FA, Holgate ST, Wardlaw AJ, Pavord ID (2002). Mast-cell infiltration of airway smooth muscle in asthma. N Engl J Med.

[6] Alwarith J, Kahleova H, Crosby L, Brooks A, Brandon L, Levin SM, Barnard ND. The role of nutrition in asthma prevention and treatment. Nutr Rev. 2020;78(11):928–38. 10.1093/nutrit/nuaa005 PMC7550896 32167552

[7] Palgan K, Bartuzi Z (2011). The role of flavonoids in asthma. Postepy Dermatol Alergol.

[8] Tarraf H, Aydin O, Mungan D, Albader M, Mahboub B, Doble A (2018). Prevalence of asthma among the adult general population of five Middle Eastern countries: results of the SNAPSHOT program. BMC Pulm Med.

[9] Heidarnia M, Entezari A, Moein M, Mehrabi Y, Pourpak Z (2007). Prevalence of asthma symptom in Iran: a meta-analysis. Res Med.

[10] McKeever TM, Britton J (2004). Diet and asthma. Am J Respir Crit Care Med.

[11] Upham JW, Chung LP (2018). Optimising treatment for severe asthma. Med J Aust.

[12] Wechsler ME (2009). Managing asthma in primary care: putting new guideline recommendations into context. Mayo Clin Proc.

[13] Agostini BA, Collares KF, Costa FDS, Correa MB, Demarco FF (2019). The role of asthma in caries occurrence - meta-analysis and meta-regression. J Asthma.

[14] Anjomshoaa I, Cooper ME, Vieira AR (2009). Caries is associated with asthma and epilepsy. Eur J Dent.

[15] Hamid S, Elhassan F, Hassan A (2015). Dental caries in 3-12-year-old Sudanese children with bronchial asthma. J Dent Res Rev.

[16] Flexeder C, Kabary Hassan L, Standl M, Schulz H, Kuhnisch J (2020). Is there an association between asthma and dental caries and molar incisor Hypomineralisation?. Caries Res.

[17] Rezende G, Dos Santos NML, Stein C, Hilgert JB, Faustino-Silva DD (2019). Asthma and oral changes in children: associated factors in a community of southern Brazil. Int J Paediatr Dent.

[18] Markovic D, Peric T, Sovtic A, Minic P, Petrovic V. [Oral health in children with asthma]. Srp Arh Celok Lek 2015;143(9–10):539–544. 10.2298/sarh1510539m 26727860

[19] O’Sullivan EA, Curzon ME (1998). Drug treatments for asthma may cause erosive tooth damage. BMJ..

[20] Storhaug K (1985). Caries experience in disabled pre-school children. Acta Odontol Scand.

[21] Meldrum AM, Thomson WM, Drummond BK, Sears MR (2001). Is asthma a risk factor for dental caries? Finding from a cohort study. Caries Res.

[22] Ersin NK, Gulen F, Eronat N, Cogulu D, Demir E, Tanac R (2006). Oral and dental manifestations of young asthmatics related to medication, severity and duration of condition. Pediatr Int.

[23] McDerra E, Pollard M, Curzon M (1998). The dental status of asthmatic British school children. Pediatr Dent.

[24] Emslie RD, Massler M, Zwemer JD (1952). Mouth breathing. I. Etiology and effects; a review. J Am Dent Assoc.

[25] Brigic A, Kobaslija S, Zukanovic A (2015). Cariogenic potential of inhaled anti-asthmatic drugs. Med Arch.

[26] Richardson WS, Wilson MC, Nishikawa J, Hayward RSA (1995). The well-built clinical question: a key to evidence-based decisions. ACP J Club.

[27] Stang A (2010). Critical evaluation of the Newcastle-Ottawa scale for the assessment of the quality of nonrandomized studies in meta-analyses. Eur J Epidemiol.

[28] Amirabadi F, Khosravi M (2013). Comparison between asthmatic and healthy children in ECC frequency. J Mashhad Dent Sch.

[29] Bahrololoomi Z, Bemanian M, Ghafourifard R, Ahmadi B (2016). Evaluation and comparison of DMFT in asthmatic and non-asthmatic 6-12 year old children in Yazd. J Shaeed Sdoughi Univ Med Sci Yazd.

[30] Bahrololoomi Z, Bemanian MH, Ghaffourifard R, Ahmadi B (2018). Effect of inhaled medication on dental caries index in asthmatic children. Allergol Immunopathol.

[31] Hassanpour K, Tehrani H, Goudarzian M, Beihaghi S, Ebrahimi M, Amiri P (2019). Comparison of the frequency of dental caries in asthmatics children under treatment with inhaled corticosteroids and healthy children in Sabzevar in 2017-2018. Electron J Gen Med.

[32] Ehsani S, Moin M, Meighani G, Pourhashemi SJ, Khayatpisheh H, Yarahmadi N (2013). Oral health status in preschool asthmatic children in Iran. Iran J Allergy Asthma Immunol.

[33] Khalilzadeh S, Salamzadeh J, Salem F, Salem K, Vala MH (2007). Dental caries-associated microorganisms in asthmatic children. Tanaffos..

[34] Katayoun S, Farzaneh S, Soheila K, Mojdeh H-V, Jamshid S (2009). Caries status in asthmatic children receiving anti-asthma inhalers. J Dent Sch.

[35] Salem K, Hamidiaval SH, Etezadkeyhani P, Lotfi G, Aghaee S (2018). Evaluation of association between asthmatic children and molar-incisor hypomineralization lesion and caries. Res Dent Sci.

[36] Ghasempour MMI, Hosaininia K (2005). Dental health status in asthmatic children. J Isfahan Dent Sch.

[37] Alavaikko S, Jaakkola MS, Tjaderhane L, Jaakkola JJ (2011). Asthma and caries: a systematic review and meta-analysis. Am J Epidemiol.

[38] Kargul B, Tanboga I, Ergeneli S, Karakoc F, Dagli E (1998). Inhaler medicament effects on saliva and plaque pH in asthmatic children. J Clin Pediatr Dent.

[39] Wogelius P, Poulsen S, Sorensen HT (2004). Use of asthma-drugs and risk of dental caries among 5 to 7 year old Danish children: a cohort study. Community Dent Health.

[40] Brigic A, Kobaslija S, Zukanovic A (2015). Antiasthmatic inhaled medications as Favoring factors for increased concentration of Streptococcus Mutans. Mater Socio Med.

[41] Lima LRS, Pereira AS, de Moura MS, Lima CCB, Paiva SM, de Deus LFA (2019). Preterm birth and asthma is associated with hypomineralized second primary molars in preschoolers: a population-based study. Int J Paediatr Dent.

[42] Vejdani J, Zahiri Sorouri Z, Emami A (2010). Survey the relationship between the type of delivery and enamel defects of the first permanent molars. J Guilan Univ Med Sci.

[43] Kharitonov SA, Yates D, Robbins RA, Logan-Sinclair R, Shinebourne EA, Barnes PJ (1994). Increased nitric oxide in exhaled air of asthmatic patients. Lancet.

[44] Schmidt-Weber CB (2006). Gene expression profiling in allergy and asthma. Chem Immunol Allergy.

[45] Ramasamy A, Kuokkanen M, Vedantam S, Gajdos ZK, Couto Alves A, Lyon HN (2012). Genome-wide association studies of asthma in population-based cohorts confirm known and suggested loci and identify an additional association near HLA. PLoS One.

[46] Ergoz N, Seymen F, Gencay K, Tamay Z, Deeley K, Vinski S (2014). Genetic variation in Ameloblastin is associated with caries in asthmatic children. Eur Arch Paediatr Dent.

[47] Simpson A, John SL, Jury F, Niven R, Woodcock A, Ollier WE (2006). Endotoxin exposure, CD14, and allergic disease: an interaction between genes and the environment. Am J Respir Crit Care Med.

[48] Yildiz G, Ermis RB, Calapoglu NS, Celik EU, Turel GY (2016). Gene-environment interactions in the Etiology of dental caries. J Dent Res.

[49] Grzela K, Litwiniuk M, Zagorska W, Grzela T (2016). Airway remodeling in chronic obstructive pulmonary disease and asthma: the role of matrix metalloproteinase-9. Arch Immunol Ther Exp.

[50] Deeley K, Letra A, Rose EK, Brandon CA, Resick JM, Marazita ML (2008). Possible association of amelogenin to high caries experience in a Guatemalan-Mayan population. Caries Res.

[51] Patir A, Seymen F, Yildirim M, Deeley K, Cooper ME, Marazita ML (2008). Enamel formation genes are associated with high caries experience in Turkish children. Caries Res.

[52] Shimizu T, Ho B, Deeley K, Briseno-Ruiz J, Faraco IM, Schupack BI (2012). Enamel formation genes influence enamel microhardness before and after cariogenic challenge. PLoS One.

[53] Garg S, Gupta S, Prakash K, Bhatnagar P (1991). Clinical ventilatory functions and immunological studies in bronchial asthma. J Indian Med Assoc.

[54] Black PN, Scicchitano R, Jenkins CR, Blasi F, Allegra L, Wlodarczyk J (2000). Serological evidence of infection with chlamydia pneumoniae is related to the severity of asthma. Eur Respir J.

[55] Aguilera Galaviz LA, Premoli G, Gonzalez A, Rodriguez RA (2005). Caries risk in children: determined by levels of mutans streptococci and Lactobaccilus. J Clin Pediatr Dent.

[56] Tahir F, Hafeez F (2018). Oral health in asthmatics: a review. J Dent Oral Care Med.

[57] Hujoel PP (2013). Vitamin D and dental caries in controlled clinical trials: systematic review and meta-analysis. Nutr Rev.

[58] Heidari A, Seraj B, Shahrabi M, Maghsoodi H, Kharazifard MJ, Zarabian T (2016). Relationship between different types and forms of anti-asthmatic medications and dental caries in three to 12 year olds. J Dent.

[59] Reddy DK, Hegde AM, Munshi AK (2003). Dental caries status of children with bronchial asthma. J Clin Pediatr Dent.

[60] Shulman JD, Taylor SE, Nunn ME (2001). The association between asthma and dental caries in children and adolescents: a population-based case-control study. Caries Res.

[61] Santos NC, Jamelli S, Costa L, Baracho Filho C, Medeiros D, Rizzo JA (2012). Assessing caries, dental plaque and salivary flow in asthmatic adolescents using inhaled corticosteroids. Allergol Immunopathol (Madr).

